# Insights from a workshop on climate-resilience in health systems in Japan and Germany: strategies to counteract social isolation and heat health risks for older adults

**DOI:** 10.1265/ehpm.26-00050

**Published:** 2026-08-29

**Authors:** Alina Herrmann, Asuna Arai, Xerxes Seposo, Ingo Meyer, Mustakim, Beate Sigrid Müller, Kayo Ueda

**Affiliations:** 1 Institute of General Medicine, Medical Faculty and University Hospital, University of Cologne, Cologne, Germany; 2 Heidelberg Institute of Global Health, Medical Faculty and University Hospital, Heidelberg University, Heidelberg, Germany; 3 Department of Hygiene, Graduate School of Medicine, Hokkaido University, Sapporo, Japan; 4 PMV forschungsgruppe, Medical Faculty and University Hospital, University of Cologne, Cologne, Germany

**Keywords:** Older adults, Elderly, Social isolation, Social network, Heat, Heat wave, Climate change, Extreme weather events, Resilience, Adaptation

## Abstract

Climate change leads to adverse health effects globally, including increased heat-related mortality of the elderly. Besides physiological factors of aging and comorbidities, social isolation is one risk factor associated with adverse health effects of heat. In 2025, the Working Group Environmental Health at Hokkaido University and the Institute of General Practice from University of Cologne held a research workshop to explore joint solutions for climate-resilience in super-aged nations like Germany and Japan. The three-day interactive workshop was held at Hokkaido University, Sapporo, Japan and included a hybrid symposium with external experts as well as intensive exchange between the research groups. Jointly addressing social isolation and heat health risks, was identified as a promising way to increase health systems resilience in times of societal and environmental crisis, especially in super-aged countries. This commentary narratively summarizes the current evidence on interventions targeting social isolation and heat health risks, finding that current interventions aim to protect socially isolated older adults during heat waves, but do not intend to alleviate both, social isolation and heat health risks at a broader scale. Based on the discussions in the workshop this commentary proposes future research opportunities, entailing family-based as well as community-based approaches, which foster social connection throughout the year and are particularly triggered during hot days and heat waves. Further research is needed to co-develop and evaluate such interventions with older adults and their families and communities to increase resilience of societies and health systems.

## Graphical Abstract

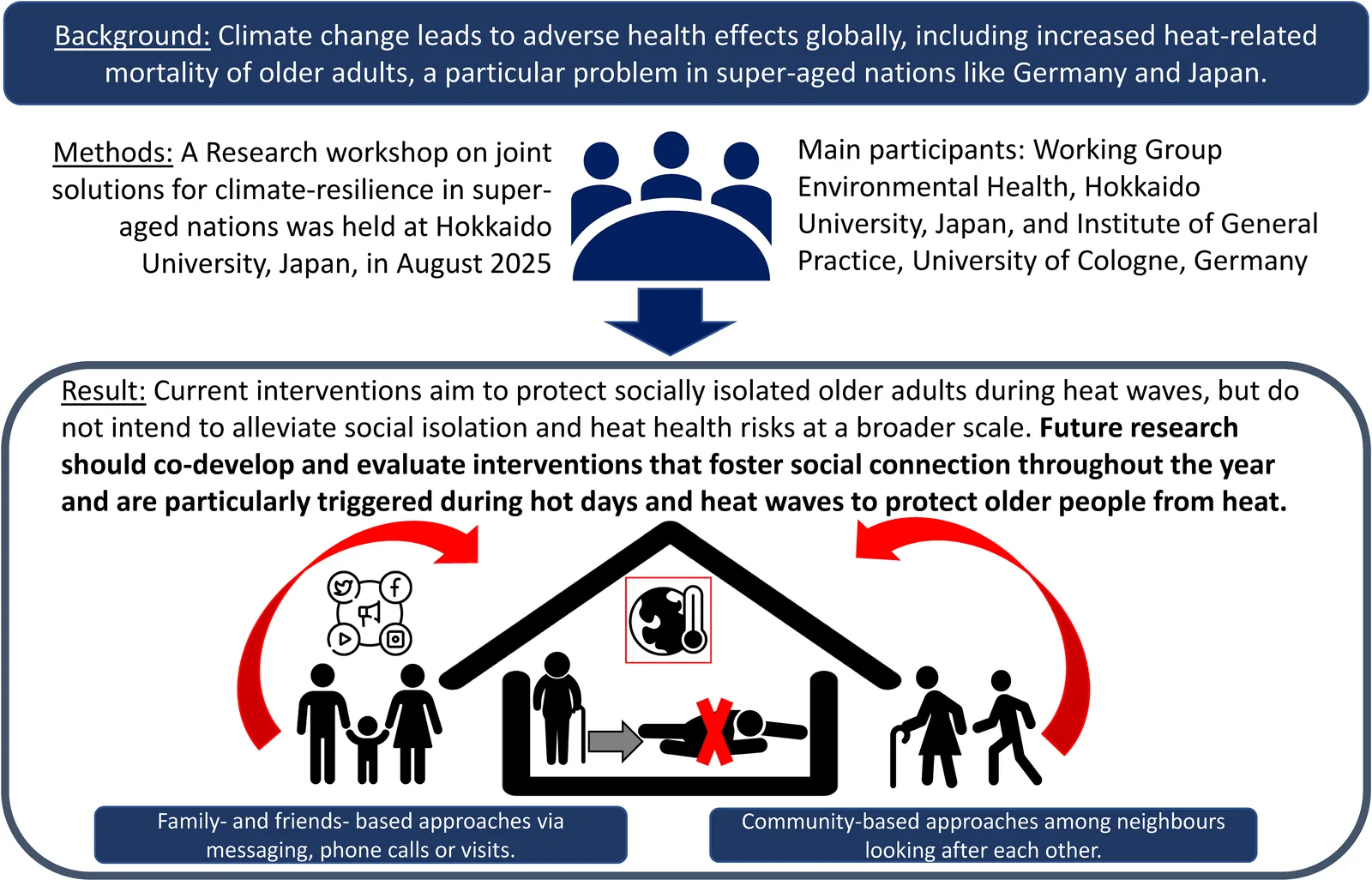

## Background

Climate change is increasingly leading to adverse health effects globally. In the latest Lancet Countdown Report on Health and Climate Change, twelve out of the twenty health-related climate indicators broke new records [1]: In 2020–2021, global heat-related mortality reached 546,000 deaths annually, 63.2% more than the 1990–1999 baseline [1]. Vulnerable populations, like children, the elderly or outdoor workers are at particular risk. In 2024, adults older than 65 years had a 304%-increased exposure to heatwaves, with an average of 20.8 heatwave days per person, compared to the 1986–2005 baseline average [1]. Several factors contribute to higher vulnerability of the elderly, such as decreased thermo-regulative capacity, comorbidities and social isolation. By 2050, the population aged over 65 is expected to double, and the population over 80 years is expected to triple compared to 2020 globally [2]. Germany and Japan are two super-aged societies, experiencing increasing heat-exposure. Both countries have similar health care systems for historic reasons [3]. In August 2025 the Working Group Environmental Health at Hokkaido University and the Institute of General Practice from University of Cologne held a research workshop to explore joint solutions for climate-resilience in super-aged nations, with a special regard for heat health risks.

## Workshop and symposium

The three-day interactive workshop was held at Hokkaido University, Sapporo, Japan. After the presentation of country profiles highlighting population ageing, the increasing number of older adults living alone and current heat-health prevention approaches in both Japan and Germany, current research work was discussed and participants visited an elderly care home in Sapporo. One afternoon a public hybrid symposium was held with over 80 participants with expertise in public health, policy and clinical patient care. At the symposium, presentations covered Japanese government’s strategy on heat health protection, the role of civil society for health-related climate action in Germany, community-based interventions for heat protection, dealing with extreme weather events and approaches to increasing sustainability and heat resilience in drug therapy. Throughout the symposium and workshop a recurring theme was social isolation of elderly. Participants highlighted that social isolation among elderly is a common phenomenon in both countries. For instance, the share of the population, who interact daily with friends and family who live close by is much lower in Japan (42%) and Germany (55%) compared to the OECD average of 67% [4]. Importantly, workshop participants identified social isolation as a factor that may increase susceptibility to climate-related extreme weather events, including heatwaves. Participants also recognized that current heat-health interventions in both countries, such as heat alert systems and community-based outreach activities, may not sufficiently reach socially isolated older adults, despite their heightened vulnerability to extreme heat [5]. Social isolation stood out as a risk factor to be addressed in future interventions, because it is modifiable, relevant in the cultural contexts of Japan and Germany and promising co-benefits for health and well-being beyond heat protection. Through these discussions, future directions for intervention research with a particular focus on socially isolated older adults were explored. According to WHO [6], social connection lies at the core of social well-being and health, describing how people relate to and interact with one another. In contrast, social isolation, as a form of social disconnection, refers to an objective lack of social roles, relationships, or interactions. In this commentary, we primarily focus on social isolation and social connection.

## State of the evidence on social isolation and heat health risks

Social isolation has well-documented negative effects on older adults’ health and is associated with a reduced capacity to cope with climate-related emergencies. One proposed mechanism is the higher prevalence of mental health conditions among socially isolated individuals, which may impair risk perception and adaptive behavior [7]. In particular, people who are socially isolated suffer from stress, anxiety and depression, which may later on progress to mental health conditions that can impair their coping capacity during heat-related emergencies [8]. Moreover, limited monitoring of the health status, lack of assistance for protective measures and poorer management of chronic conditions may be a reason for the association of social isolation and adverse health effects of heat. In the super-aging society of South Korea, a time-series study demonstrated that heatwave-related mortality among the urban elderly population was exacerbated by higher levels of social isolation. The authors found that a one-percentile reduction in social gathering—used as an indicator of social connection—among elderly males living in highly dense areas was associated with a 2% increase in heatwave-related mortality risk (95% CI: 0.5%, 3.4%) [9].

## Interventions targeting social isolation and heat health risks

While being socially isolated has been identified as a risk factor for adverse health effects of heat, there is surprisingly little literature describing interventions, which look at joint interventions aiming to increase social connection and decrease heat related mortality. Kafeety et al. [7] argue that socially isolated persons need to be targeted specifically as they might not profit from general heat health protection strategies and because increasing social connection might be a major protective factor from adverse heat events. We found only two studies, published by a research team in Rome, which evaluated the effects of social connection measures on heat related mortality, once in a quasi-experimental study design and once in a retrospective ecological design. In the quasi-experimental study a program identifying and monitoring socially isolated elderly over 80 years of age in three districts in Rome, Italy, aimed to strengthen individual networks and social capital of the community to reduce adverse health effects of heat. Mortality outcomes of elderly within the program were compared to counterparts in the same districts, providing some evidence for a reduced mortality in those signed-up in the program [10]. In the ecological study, the same intervention was retrospectively assessed for its effects on reducing heat wave mortality [11]. It found that the incidence rate of excess death was lower in the intervention group compared to the control (adjusted incidence rate ratio 0.44, CI 0.32–0.60).

This scarcity of studies might stem from several factors. First, research on health impacts of climate change and research on aging in general, and social isolation in particular, have so far been treated as separate fields [12]. Second, both climate change and the aging of societies worldwide have accelerated dramatically over the past decade, making solutions for both urgently needed. However, due to the rapid pace and complexity of these developments, especially combined solutions are not easily available. Third, there is a general lack of studies, which rigorously evaluate the effects of heat health protection measures. This might be due to the mostly complex and non-controlled nature of measures if they are directed at e.g. whole urban areas in the context of heat health action plans (HHAPs). Several studies evaluate the effectiveness of heat health action plans, mostly finding that HHAPs can reduce heat related mortality. A recent study in Europe found, that implementation of heat protection measures could reduce heat-related excess death by 25% [13]. Swiss cities, which implemented HHAPs including buddy programs to assist individuals at risk, showed a significant decrease of heat-related mortality over time [14]. However, we don’t know of any study, which concretely assesses the effects of single interventions addressing social isolation within HHAPs.

Yet, several HHAPs include elements that increase social connectedness: Philadelphia’s HHAP in the USA incorporates “buddy systems” where the public health service trains volunteers in the neighbourhood as heat buddies for individuals at high risk, including outreach and regular check-ins during periods of heat stress. Under the French HHAP, municipalities maintain a heatwave registry of vulnerable residents, who are proactively called by local social services during heat alerts to assess needs and provide tailored advice, reinforcing social contact and monitoring for signs of heat-related distress. The public health authority of the city of Cologne in Germany offers a free heat hotline, where older adults and other vulnerable persons can register and receive personalized advice on coping with heat before and during the summer. In the Lazio region’s HHAP in Italy, general practitioners and district health services actively participate in surveillance and follow-up of susceptible individuals during heatwaves, including routine contact and health monitoring of older patients and chronically ill individuals. All of these examples try to protect socially isolated older adults during heat waves. However, they don’t explicitly address the social isolation itself, as they work with trained volunteers or professionals trying to alleviate missing support during heatwaves only. To achieve greater resilience in the health system in general, interventions that explicitly try to reduce heat health risks of socially isolated elderly and reduce social isolation in itself seem to be warranted. Ideally, these interventions should not require additional resources from the healthcare system (e.g., professional outreach services), but rather involve families, friends and the wider community. Such integrated approaches are needed to provide sustainable solutions, moving beyond acute crisis management to foster long-term community well-being and adaptive capacity against future climatic and social stressors. Only this way can a transformational pathway be achieved, which not only counteracts climate-related risks, but also increases health system performance, as suggested by the World Health Organizations’ vision for climate-resilient health systems [15].

## Future research opportunities

During the workshop we developed two main ideas for future research that promote social connection and resilience in the face of aging societies and climate change. **Existing relationships (1)** of elderly should be leveraged by encouraging family members, friends and neighbours to contact each other daily during heat waves. Such campaigns should also entail a transformative element trying to keep up the closer social connection beyond the summer, thus, addressing the risk factor of social isolation in itself to improve health outcomes beyond adverse health effects of heat. **New relationships (2)** within the community should be initiated within the neighborhood, so that elderly could act as reciprocal heat-buddies during the summer and extend such new relationships beyond times of extreme heat. Given the limited space of this comment, we will only detail on the first idea in the following.

Current heat phone lines may face barriers such as their high resource requirements and reluctance of some older adults to enroll in programs administered by public authorities. Because trust and routine communication are often already established within families, a family-based relationship-strengthening programme may help overcome some of these limitations. In addition to its potential role in heat prevention, this approach may also strengthen intergenerational social connection, which could yield broader benefits for individual health and community well-being [16]. At the same time, several challenges need to be considered. A key question is how family members can effectively encourage preventive behaviors among older adults. Some older adults may refrain from using air conditioners or maintaining adequate hydration during hot weather because of economic concerns or discomfort, as well as reduced heat awareness, diminished thirst sensation, cognitive or mobility impairments, and a lowered perception of heat-related risk. Long-established habits and routines can be difficult to change, particularly among older populations. Recent studies have identified key beliefs motivating heat adaptation behaviors [17] and effective strategies for heat-risk communication [18]. Thus, short educational activities on how to nudge desired behaviors, drawing on behavior change theories, would be viable way forward.

It is also necessary exploring alternative or complementary forms of communication to traditional phone calls. Digital messaging applications or other communication tools may play a supportive and more convenient role. Furthermore, cultural patterns of family interaction, heat-related habits, and attitudes toward heat prevention need to be considered across different settings like Germany and Japan. Therefore, co-designing interventions together with elderly and family members in the targeted communities is important. Comparative research between Germany and Japan could provide valuable insights into both, universal principles and context-specific strategies for heat prevention tailored to different communities.

## Conclusions

Joint strategies to counteract social isolation and heat-related health risks among older adults could be a promising way forward to strengthen health systems in general and their climate-resilience. More research co-developing appropriate interventions with elderly and their families and communities, and testing the interventions effectiveness is needed. Such research is particularly relevant for super-aged societies like Japan and Germany and its findings are likely to be applicable beyond these contexts.

## Declarations

### Availability of data and materials

Not applicable, as no primary data was included in the manuscript.

### Use of artificial intelligence

We used artificial intelligence (AI, Chat-GPT 5) for language editing of selected paragraphs of this manuscript, as none of the authors are native speakers. All changes made by AI were checked and approved by the authors and the authors take full responsibility of the manuscript.
